# Study of CD27 and CCR4 Markers on Specific CD4^+^ T-Cells as Immune Tools for Active and Latent Tuberculosis Management

**DOI:** 10.3389/fimmu.2018.03094

**Published:** 2019-01-09

**Authors:** Irene Latorre, Marco A. Fernández-Sanmartín, Beatriz Muriel-Moreno, Raquel Villar-Hernández, Sergi Vila, Maria L. De Souza-Galvão, Zoran Stojanovic, María Á. Jiménez-Fuentes, Carmen Centeno, Juan Ruiz-Manzano, Joan-Pau Millet, Israel Molina-Pinargote, Yoel D. González-Díaz, Alicia Lacoma, Lydia Luque-Chacón, Josefina Sabriá, Cristina Prat, Jose Domínguez

**Affiliations:** ^1^Servei de Microbiologia, Hospital Universitari Germans Trias i Pujol, Institut d'Investigació Germans Trias i Pujol, Barcelona, Spain; ^2^CIBER Enfermedades Respiratorias, CIBERES, Instituto de Salud Carlos III, Madrid, Spain; ^3^Departament de Genètica i Microbiologia, Universitat Autònoma de Barcelona, Barcelona, Spain; ^4^Plataforma de Citometría, Institut d'Investigació Germans Trias i Pujol, Barcelona, Spain; ^5^Unitat de Tuberculosi de Drassanes, Hospital Universitari Vall d'Hebron, Barcelona Spain; ^6^Servei de Pneumologia, Hospital Universitari Germans Trias i Pujol, Barcelona, Spain; ^7^Serveis Clínics, Unitat Clínica de Tractament Directament Observat de la Tuberculosi, Barcelona, Spain; ^8^CIBER de Epidemiología y Salud Pública, CIBERESP, Instituto de Salud Carlos III, Madrid, Spain; ^9^Servei de Pneumologia, Hospital Sant Joan Despí Moises Broggi, Sant Joan Despí, Barcelona, Spain

**Keywords:** CD4 T-cells, CD27, CCR4, flow cytometry, immunity, latent tuberculosis, tuberculosis

## Abstract

The immunological characterization of different cell markers has opened the possibility of considering them as immune tools for tuberculosis (TB) management, as they could correlate with TB latency/disease status and outcome. CD4^+^ T-cells producing IFN-γ^+^ with a low expression of CD27 have been described as an active TB marker. In addition, there are unknown homing receptors related to TB, such as CCR4, which might be useful for understanding TB pathogenesis. The aim of our study is focused on the assessment of several T-cell subsets to understand immune-mechanisms in TB. This phenotypic immune characterization is based on the study of the specific immune responses of T-cells expressing CD27 and/or CCR4 homing markers. Subjects enrolled in the study were: (i) 22 adult patients with active TB, and (ii) 26 individuals with latent TB infection (LTBI). Blood samples were drawn from each patient. The expression of CD27 and/or CCR4 markers were analyzed within CD4^+^ T-cells producing: (i) IFN-γ^+^, (ii) TNF-α^+^, (iii) TNF-α^+^IFN-γ^+^, and (iv) IFN-γ^+^ and/or TNF-α^+^. The percentage of CD27^−^ within all CD4^+^ T-cell populations analyzed was significantly higher on active TB compared to LTBI after PPD or ESAT-6/CFP-10 stimulation. As previously reported, a ratio based on the CD27 median fluorescence intensity (MFI) was also explored (MFI of CD27 in CD4^+^ T-cells over MFI of CD27 in IFN-γ^+^CD4^+^ T-cells), being significantly increased during disease (*p* < 0.0001 after PPD or ESAT-6/CFP-10 stimulation). This ratio was also assessed on the other CD4^+^ T-cells functional profiles after specific stimulation, being significantly associated with active TB. Highest diagnostic accuracies for active TB (AUC ≥ 0.91) were achieved for: (i) CD27 within IFN-γ^+^TNF-α^+^CD4^+^ T-cells in response to ESAT-6/CFP-10, (ii) CD27 and CCR4 markers together within IFN-γ^+^CD4^+^ T-cells in response to PPD, and (iii) CD27 MFI ratio performed on IFN-γ^+^TNF-α^+^CD4^+^ T-cells after ESAT-6/CFP-10 stimulation. The lowest diagnostic accuracy was observed when CCR4 marker was evaluated alone (AUC ≤ 0.77). CD27 and CCR4 expression detection could serve as a good method for immunodiagnosis. Moreover, the immunological characterization of markers/subset populations could be a promising tool for understanding the biological basis of the disease.

## Introduction

Given the limited knowledge on tuberculosis (TB) biomarkers, the study of different T-cell subsets, as well as *Mycobacterium tuberculosis* specific antigens and cytokines, are attractive options to follow in order to understand TB pathogenesis as well as the interplay between infection and disease (1–3). Usually, TB outcome is understood as a bimodal model between active TB and latent TB infection (LTBI). However, in the past years, infection has been associated with a dynamic and wide spectrum containing different latency phases (4). Furthermore, active TB is known to be a heterogeneous disease which comprises a wide range of manifestations and forms. The key to control the spread of TB is a rapid diagnosis in an early stage. However, active TB confirmation can be difficult and the commonly used test systems are still insufficient. Due to all these reasons, the development of alternative diagnostic methods remains a challenge for improving TB control. In this aspect, the immunological characterization of different cell markers has opened the possibility of considering them as immune tools for TB management, as they could correlate with cell differentiation, latency/disease status, and outcome (1, 5).

*M. tuberculosis* sensitization can be detected by tuberculin skin test (TST), classically used in LTBI diagnosis. However, this assay presents cross-reactive antigens and other proteins which could lead to false-positive results. More than a decade ago, the *in vitro* interferon (IFN)-gamma (γ) release assays (IGRAs) were introduced. These tests have been proven to be more specific and useful for LTBI diagnosis and sensitization detection than the TST. They are not affected by cross-reaction caused by BCG vaccination and/or non-tuberculous mycobacteria (NTM) infection (6–8). These techniques are based on the detection of IFN-γ released by sensitized T-cells after stimulation with specific *M. tuberculosis* antigens (ESAT-6 and CFP-10) (9). Nevertheless, both TST and IGRAs identify sensitization to the bacilli, which is translated into a detectable immune response. Therefore, they cannot discriminate between active TB and infection, nor identify those individuals with high risk of developing active TB when infected (10, 11). New biomarkers are therefore needed to improve disease immune diagnosis, providing prognostic information, assessing risk-stratification in LTBI individuals, and revealing general biological mechanisms of the pathogenesis.

CD4^+^ T-cells with a low expression of CD27 have been described as an immune biomarker of active TB disease and lung tissue destruction. The decrease of CD27 expression indicates the existence of differentiated effector T-cells which produce cytokines upon antigen encounter. This subset phenotype (CD27^−^CD4^+^ or CD27^low^CD4^+^) is specifically increased in whole blood and at the site of infection during active disease (12–15). This is due to the process known as homing, which refers to the migration of specific cell subsets to the infected tissue. Recently, a new strategy based on CD27 marker detection has been developed for active TB diagnosis (16, 17). This assay is able to discriminate between active TB and LTBI by analyzing CD27 expression on specific *M. tuberculosis* CD4^+^ T-cells that respond secreting IFN-γ. This new approach, assesses the ratio of the median fluorescence intensity (MFI) between CD27 on CD4^+^ T-cells and CD27 on specific T-cells in response to PPD or ESAT-6/CFP-10 antigens. There are still other novel and potential homing markers to explore that might be useful tools for understanding TB pathogenesis and improving diagnosis. For example, the chemokine receptor CCR4, which is considered a homing marker that could be expressed on several cells of the immune system including T helper type 1 (Th1) cells. It is known that T-cells expressing CCR4 surface marker are recruited in inflammatory sites (18). Some evidence suggest that the induction of CCR4 expression is associated with the migration of CD4^+^ T-cells into the lungs, indicating that this homing marker could play a protective role in the immunity against some respiratory pathogens (19, 20). Together, these findings open the possibility for new studies on the development of novel strategies for TB management and understanding the different mechanisms against the disease. In the present study, we focus on the assessment of several T-cell subsets in order to characterize different TB latency/disease immune-mechanisms. This immune characterization could allow the development of new strategies for TB management based on the study of the immune response of T-cells expressing CD27 and/or CCR4 markers in patients with active TB and LTBI individuals.

## Materials and Methods

### Study Population and Inclusion Criteria

For this study we enrolled subjects with active TB or LTBI suspicion, who attended the four following centers located in Barcelona (Spain): Hospital Germans Trias i Pujol, Unitat de Tuberculosi Vall d'Hebron-Drassanes, Serveis Clínics-Unitat Clínica de Tractament Directament Observat de la Tuberculosi and Hospital Sant Joan Despí Moises Broggi. A total of 16 mL of blood per patient were drawn in CPT tubes (BD Biosciences, San Jose, CA, USA). Blood was directly sent to the Institut d'Investigació Germans Trias i Pujol for peripheral blood mononuclear cells (PBMCs) isolation and cytometry testing.

Subjects enrolled in the study were classified as: (i) adult patients with active TB (pulmonary or extrapulmonary) with a positive culture and/or PCR for *M. tuberculosis*. Patients were enrolled within the first 4 weeks of starting anti-TB therapy; and (ii) individuals with LTBI enrolled during contact tracing studies or LTBI screenings. In this group, LTBI was defined based on a positive TST and/or IGRAs in the absence of clinical symptoms and radiological signs compatible with active TB. Chemoprophylaxis was prescribed in all of these subsets, being all of them enrolled during the first 4 weeks of preventive therapy.

TST was performed according the Mantoux technique using two tuberculin units of PPD RT23 (Statens Serum Institut, Copenhagen, Denmark), and was evaluated within 48–72 h. According to the Spanish Pulmonology and Thoracic Surgery Society guidelines, a TST ≥ 5 mm was considered positive (21, 22). T-SPOT.TB (Oxford Immunotec, Abingdon, UK) and QuantiFERON-TB Gold In-Tube (QFN-G-IT; Qiagen, Düsseldorf, Germany) were performed and interpreted according to the manufacturer's instructions provided in the kits.

### PBMCs Isolation, Preservation, and Stimulation

PBMCs were isolated using CPT tubes (BD Biosciences). Afterwards, cells were cryopreserved and stored in liquid nitrogen for later flow cytometry analyses. Cryopreserved PBMCs were thawed and rested during 2 h in a humidified incubator at 37°C with 5% CO_2_ in RPMI 1,640 medium (Biowest, Nuaillé, France) containing 10% of heat-inactivated fetal calf serum (FCS) with benzonase (Sigma, St. Louis, MO, USA; final concentration 10 U/mL). PBMCs from each patient were stimulated overnight at 37°C with 5% CO_2_ with the recombinant proteins ESAT-6/CFP-10 (Lionex Diagnostics and Therapeutics, Braunschweig, Germany; final concentration 2 μg/mL for each antigen) and PPD (Statens Serum Institut, Copenhagen, Denmark; final concentration 10 μg/mL). The staphylococcal enterotoxin B (SEB; Sigma; final concentration 2.5 μg/mL) was used as a positive control. A negative control without stimulation was also included. Cells were also co-stimulated with anti-CD28 and anti-CD49d monoclonal antibodies (BD Bioscience; final concentration 2 μg/mL each). After 2 h of incubation, Brefeldin A (BFA; Sigma; final concentration 3 μg/mL) was added into the culture media to inhibit the intracellular vesicular transport. Then, PBMCs were left in the incubator overnight.

### Surface and Intracellular Staining

After stimulation, PBMCs were stained with the following surface antibodies: anti-CD4 BV786 (BD Bioscience; clone SK3), anti-CD3 PerCP (BioLegend, San Diego, CA, USA; clone SK7), anti-CD27 BV605 (BD Bioscience; clone L128), anti-CCR4 PE-CF594 (BD Bioscience; clone 1G1), and anti-CD8 BV510 (BD Bioscience; clone SK1). A viability marker was also used to exclude dead cells (LIVE/DEAD, Near-IR fluorescent reactive dye; Thermo Fisher, Waltham, MA, USA). For intracellular staining, PBMCs were fixed/permeabilized (IntraStain; Dako, Santa Clara, CA, USA) and then stained with anti-IFN-γ APC (BD Bioscience; clone B27) and anti-TNF-α PE-Cy7 (BD Bioscience; clone Mab11). Markers detection was performed in a BD LSRFortessa flow cytometer (BD Bioscience). A total of 100.000 alive CD3^+^ T-cells were acquired within 2–3 h after staining.

### Flow Cytometry and Data Analysis

Aggregated cells were excluded by gating on the diagonal that appears with *forward scatter* (FSC)-H and FSC-A characteristics. Then, lymphocytes were selected according to their FSC-A and *side scatter* (SSC)-A. CD4^+^ and CD8^+^ T-cells were gated based on alive CD3^+^ T-cells. The gating strategy is represented in Figure S1 in Supplementary Material. Specific IFN-γ and/or TNF-α secretion was analyzed on CD4^+^ and CD8^+^ T-cells. The expression of CD27 and/or CCR4 markers were studied within the following populations after PPD or ESAT-6/CFP-10 stimulation: (i) IFN-γ^+^CD4^+^ T-cells, (ii) TNF-α^+^CD4^+^ T-cells, and (iii) IFN-γ^+^TNF-α^+^CD4^+^ T-cells. In addition, a boolean analysis was performed on CD4^+^ T-cells producing IFN-γ^+^and/orTNF-α^+^ in order to characterize the expression of CD27 and/or CCR4 on T-cells producing any cytokine. To assess the expression of CD27 and/or CCR4 homing markers, the frequency of cytokine production after specific stimulation was defined as positive when it was twice the amount when compared to its negative control (unstimulated sample). Fluorescence Minus One (FMO) controls were included in each experiment to set up the gates. A ratio based on CD27 MFI was also calculated as suggested by Portevin et al. (16). This ratio is performed measuring the MFI of CD27 marker on CD4^+^ T-cells over the MFI of CD27 marker on IFN-γ^+^CD4^+^ specific T-cells. This ratio based on CD27 MFI was also studied in the other T-cells phenotypes (TNF-α^+^CD4^+^; IFN-γ^+^TNF-α^+^CD4^+^; and IFN-γ^+^and/orTNF-α^+^CD4^+^ T-cells). Results comparing percentages of CD27^−^ and/or CCR4^+^ T-cells, as well as CD27 MFI ratios between groups were performed using the two-tailed Mann-Whitney *U*-test for pairwise comparisons. Differences were considered statistically significant when a *p*-value was < 0.05. Correlations between the percentage of CD27^−^ T-cells and CD27 MFI ratio were calculated using the two-tailed non-parametric Spearman test. Receiver operating characteristic (ROC) analysis and areas under the curve (AUC) were calculated in order to assess the accuracy of the different biomarkers for TB diagnosis. Flow cytometry data was analyzed using BD FACSDiva software (BD Bioscience). Graphical representation is based on GraphPad Prism version 4 (GraphPad Software, Inc, San Diego, CA).

## Results

### Patient Characteristics

A total of 48 subjects were enrolled in the study: (i) 22 active pulmonary and extrapulmonary TB patients with *M. tuberculosis* culture confirmation, and (ii) 26 individuals with LTBI. Demographical and clinical characteristics are detailed in Table 1. Overall, 62.5 (30/48) were men and 37.5% (18/48) women. The mean age (years) ± standard deviation (SD) was 43.54 ± 16.06.

**Table 1:** Demographic and clinical characteristics of the participants regarding the study group.

**VARIABLES**	**Active TB**	**LTBI**
Participants, *n*	22	26
Mean age, years ± SD	38.68 ± 15.35	47.65 ± 15.77
Male gender, *n*(%)	18 (81.8)	12 (46.2)
**POSITIVE PCR**, ***n*****(%)**		
Positive	10 (45.4)	–
Negative	4 (18.2)	–
Unknown	8 (36.4)	–
**TYPE of TB**		
Pulmonary	18 (81.8)	–
Extrapulmonary^a^	4 (18.2)	–
**ANTI-TB TREATMENT**, ***n*****(%)**		
Before starting treatment	3 (13.6)	–
After starting treatment (< 1month)	19 (86.4)	–
Not prescribed	–	26 (100)
Mean time of anti-TB treatment, days ± SD	15.14 ± 9.77	–
**CHEMOPROPHYLAXIS**, ***n*****(%)**		
Before starting treatment	–	2 (7.7)
After starting treatment (< 1month)	–	24 (92.3)
Not prescribed	22 (100)	–
Mean time of chemoprophylaxis, days ± SD	–	18.6 ± 8.06
**PPD CYTOMETRY RESPONDERS**, ***n*****(%)**^**b**^		
IFNγ^+^CD4^+^ T cells	22 (100)	26 (100)
TNF-α^+^CD4^+^ T-cells	22 (100)	26 (100)
TNF-α^+^IFN-γ ^+^CD4^+^ T-cells	22 (100)	26 (100)
IFNγ^+^and/orTNF-α^+^CD4^+^ T-cells	22 (100)	26 (100)
**ESAT-6/CFP-10 CYTOMETRY RESPONDERS**, ***n*****(%)**^**b**^		
IFNγ^+^CD4^+^ T cells	21 (95.5)	21 (80.8)
TNF-α^+^CD4^+^ T-cells	22 (100)	25 (96.2)
TNF-α^+^IFN- γ ^+^CD4^+^ T-cells	21 (95.5)	21 (80.8)
IFNγ^+^and/orTNF-α^+^CD4^+^ T-cells	22 (100)	25 (96.2)

a *Pleural TB (n = 2), ganglionar TB (n = 1), and pericardical TB (n = 1)*.

b *Number of individuals with a positive CD4^+^ T-cell response to the specified antigen. The frequency of the response to any cytokine after specific stimulation was defined as positive when it was twice the amount when compared to its negative control (unstimulated sample)*.

*TB, tuberculosis; LTBI, latent tuberculosis infection; SD, standard deviation; PPD, purified protein derivative*.

### Cytokine Profile of *M. tuberculosis* Specific CD4^+^ and CD8^+^ T-Cell Response in Active TB and LTBI Individuals

**Figure 1:**
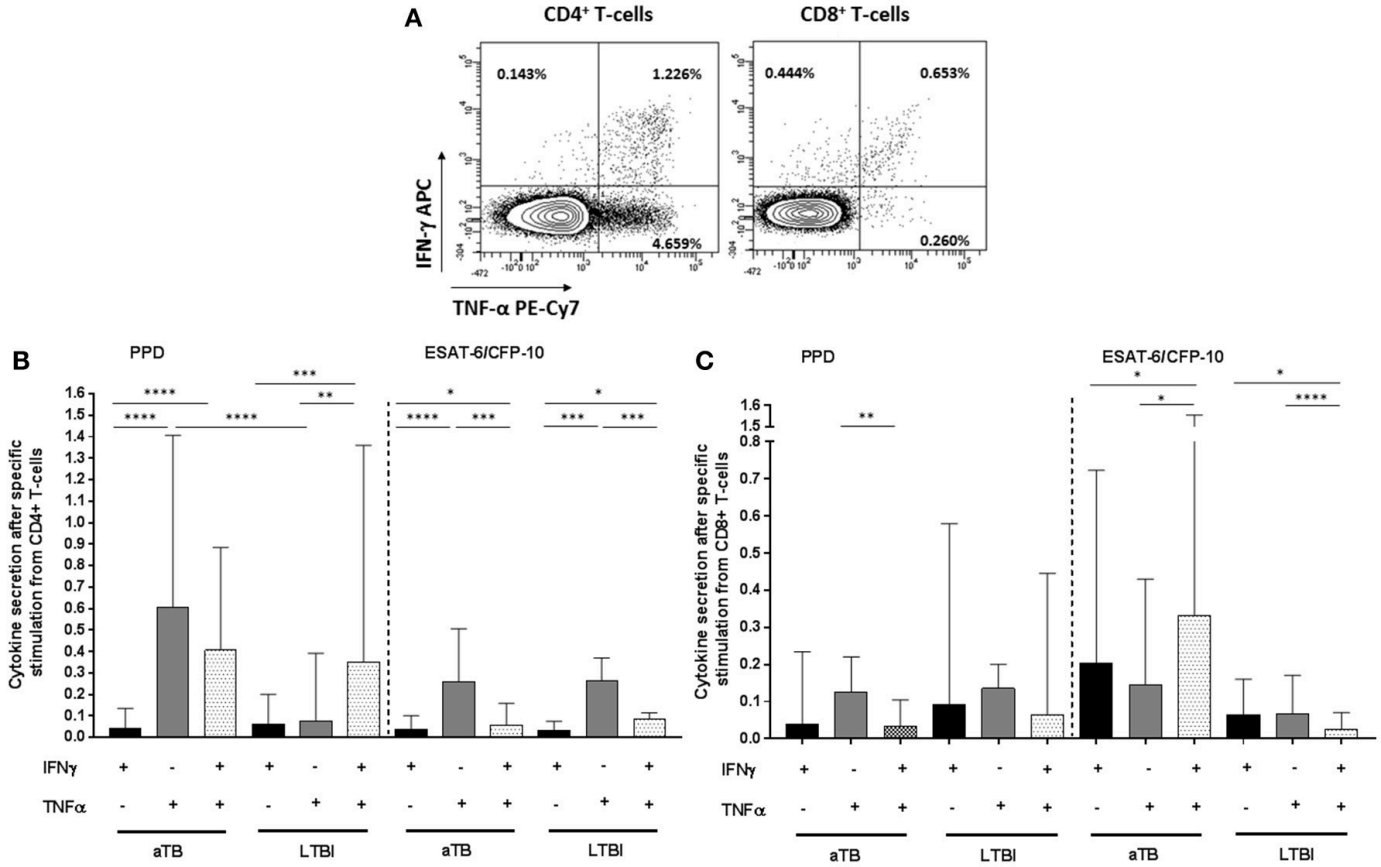
CD4^+^/CD8^+^ T-cells specific cytokine secretion phenotype regarding active TB or LTBI. **(A)** Dot plots from one active TB representative donor showing the expression of CD4^+^ and CD8^+^ T-cells producing IFN-γ and/or TNF-α after PPD stimulation. The frequency of the different cytokines production profile from the total CD4^+^ and CD8^+^ T-cells is indicated in each dot plot. **(B)** CD4^+^ T-cells and **(C)** CD8^+^ T-cells cytokine secretion after PPD or ESAT6/CFP10 stimulation. Black bars represent T-cells which only produce IFN-γ cytokine. Gray bars correspond to T-cells which only produce TNF-α. Dotted bars represent T-cells producing both IFN-γ and TNF-α cytokines. Bars depict medians with interquartile ranges. Differences between conditions were calculated using the two-tailed Mann-Whitney *U*-test. Only significant differences are represented in the graphs. *p < 0.05, **p < 0.01, ***p < 0.001, ****p < 0.0001. aTB, active TB; LTBI, latent tuberculosis infection.

To better define the specific *M. tuberculosis* responses in active TB and LTBI individuals, IFN-γ and/or TNF-α cytokines were measured on CD4^+^/CD8^+^ T-cells after PPD or ESAT-6/CFP-10 stimulation (Figure 1A). All individuals included in this study were responsive to SEB positive control in the cytometry assays.

Regarding CD4^+^ T-cells functional profile after PPD stimulation, cells producing only IFN-γ were significantly lower compared to cells producing only TNF-α^+^ or IFN-γ^+^/TNF-α^+^ simultaneously in active TB patients (*p* < 0.0001 for IFN-γ^+^ vs. TNF-α^+^ and *p* < 0.0001 for IFN-γ^+^ vs. IFN-γ^+^/TNF-α^+^) and LTBI individuals (*p* = 0.0001 for IFN-γ^+^ vs. IFN-γ^+^/TNF-α^+^). This was also observed after ESAT-6/CFP-10 stimulation in disease patients (*p* < 0.0001 for IFN-γ^+^ vs. TNF-α^+^ and *p* = 0.035 for IFN-γ^+^ vs. IFN-γ^+^/TNF-α^+^) and LTBI (*p* < 0.0001 for IFN-γ^+^ vs. TNF-α^+^ and *p* = 0.043 for IFN-γ^+^ vs. IFN-γ^+^/TNF-α^+^). Interestingly, cells that only produced TNF-α after PPD stimulation were significantly increased in active TB in comparison with LTBI (*p* < 0.0001). This difference was not observed after ESAT-6/CFP-10 specific stimulation (Figure 1B).

Regarding CD8^+^ T-cells, specific cytokines responses were detectable in active TB patients and LTBI individuals, indicating that this T-cell population is also abundant in the immune response against *M. tuberculosis*. After PPD stimulation, cells which produced only TNF-α were predominant in active TB patients (*p* = 0.009 for TNF-α^+^ vs. IFN-γ^+^/TNF-α^+^). This phenotype was not observed for LTBI individuals. In addition, although differences were not significant, cytokine response frequency after ESAT-6/CFP-10 stimulation was higher in active TB vs. LTBI (Figure 1C).

### Expression of CD27 and/or CCR4 Markers Regarding the Clinical Status

**Figure 2:**
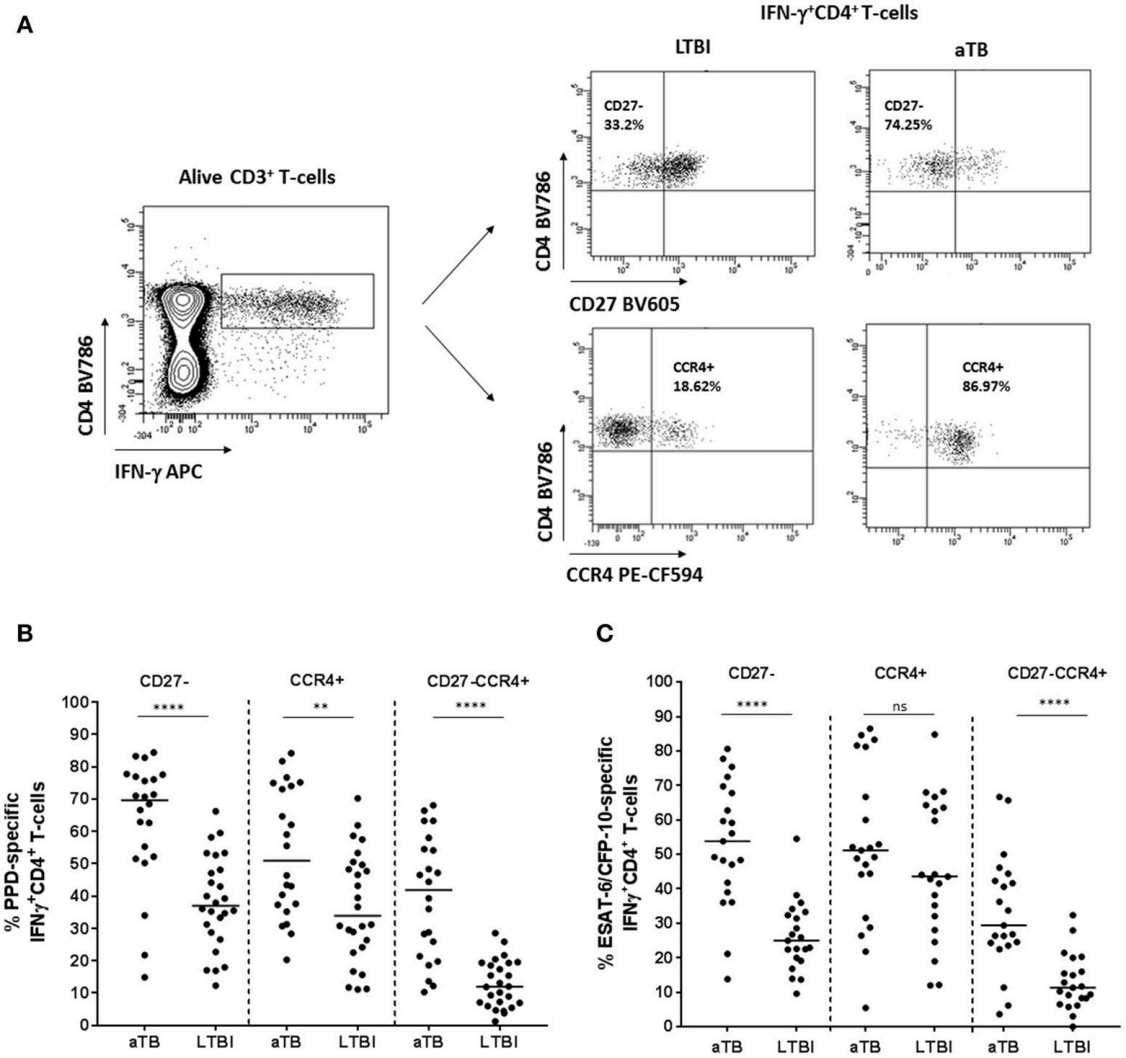
CD27^−^ and/or CCR4^+^ phenotype from CD4^+^IFN-γ^+^ specific T-cells in patients with active TB and LTBI individuals. **(A)** Representative example from an active TB and a LTBI individual showing the strategy for determining CD27^−^ or CCR4^+^ T-cells after PPD stimulation. CD27 and CCR4 expression was analyzed within the specific IFN-γ^+^CD4^+^ T-cells population after PPD or ESAT-6/CFP-10 stimulation. A negative control without stimulation was also included in the analysis for each patient enrolled in the study. Fluorescence Minus One (FMO) controls were included in each experiment for setting up gates. **(B)** Percentage of PPD or **(C)** ESAT6/CFP10 specific CD27^−^, CCR4^+^, and CD27^−^CCR4^+^ within IFN-γ^+^CD4^+^ specific T-cells. Horizontal lines represent medians. Differences between conditions were calculated using the two-tailed Mann-Whitney *U*-test. **p < 0.01, ****p < 0.0001. ns, non-significant. aTB, active TB; LTBI, latent tuberculosis infection.

The percentage of CD27^−^ and/or CCR4^+^ surface homing markers was studied on active TB and LTBI within the IFN-γ^+^CD4^+^ T-cells subset after *M. tuberculosis* specific stimulation (Figure 2A). When the expression of CD27 and CCR4 was analyzed separately, the proportion of CD27^−^ or CCR4^+^ within IFN-γ^+^CD4^+^ T-cells was significantly higher in active TB when compared with LTBI in response to PPD (*p* < 0.0001 for CD27^−^ and *p* = 0.006 for CCR4^+^) or ESAT-6/CFP-10 recombinant proteins (*p* < 0.0001 for CD27^−^), with the exception of CCR4 marker in response to ESAT-6/CFP-10, where no statistical significance was obtained. In addition, both surface T-cell markers were analyzed together (CD27^−^CCR4^+^ phenotype within IFN-γ^+^CD4^+^ T-cell compartment). The proportion of CD27^−^CCR4^+^IFN-γ^+^CD4^+^ T-cells was significantly associated with active TB (*p* < 0.0001 after PPD or ESAT-6/CFP-10 stimulation; Figures 2B,C) and reduced the overlapping between the two clinical status after PPD stimulation. These findings could indicate that the loss of CD27 and the increase of CCR4 markers could be associated with *M. tuberculosis* uncontrolled replication. In our study, active TB patients were recruited within the 4 weeks of starting therapy. In order to explore if these firsts weeks after treatment initiation influenced the expression of CD27 and/or CCR4 markers, we performed a Spearman test correlation. No significant correlation was observed between days of treatment (within the 4 weeks of starting therapy) and the percentage of CD27^−^ and/or CCR4^+^ within IFN-γ^+^CD4^+^ T-cells in response to PPD (Figure S2A in Supplementary Material) or ESAT-6/CFP-10 (Figure S2B in Supplementary Material).

CD27 and/or CCR4 markers were further characterized on CD4^+^ T-cells producing: (i) TNF-α^+^, (ii) TNF-α^+^IFN-γ^+^, and (iii) IFN-γ^+^ and/or TNF-α^+^ (Boolean analysis) after *M. tuberculosis* specific stimulation. The proportion of CD27^−^, CCR4^+^, and CD27^−^CCR4^+^ T-cells within these three subsets was significantly higher in active TB patients compared to LTBI individuals in response to PPD or ESAT-6/CFP-10 specific stimulation (Figure S3 in Supplementary Material), with the exception of CCR4 marker in response to ESAT-6/CFP-10, where no statistical significance was obtained. Furthermore, a significant positive correlation was observed on CD27^−^ expression between antigen-specific IFN-γ^+^CD4^+^ and TNF-α^+^CD4^+^ T-cells (Figure S4A in Supplementary Material) or TNF-α^+^IFN-γ^+^CD4^+^ T-cells (Figure S4B in Supplementary Material).

### CD27 MFI Ratio Analysis

**Figure 3:**
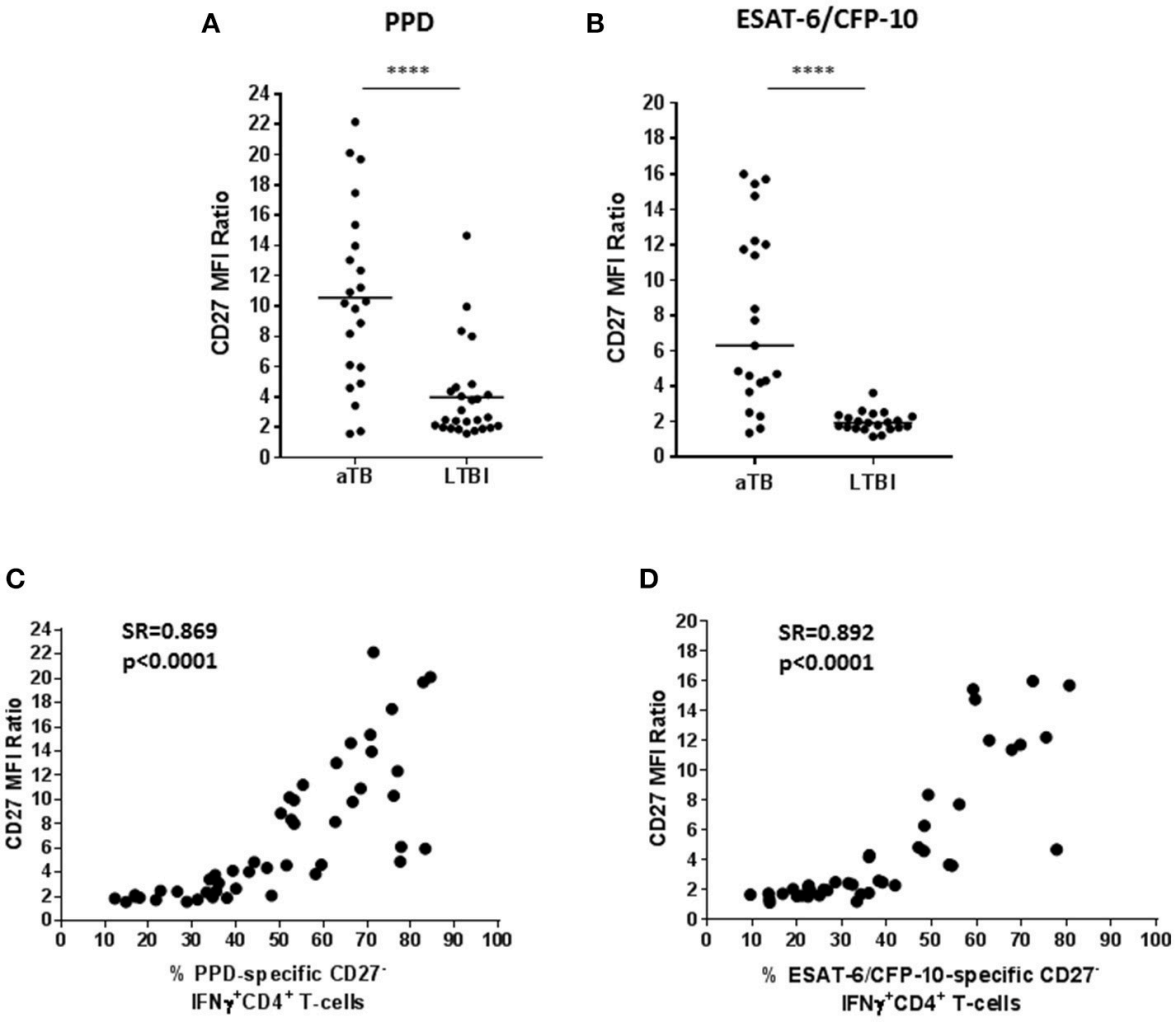
CD27 MFI ratio according to the study group. A ratio based on CD27 MFI was calculated after specific stimulation as suggested by Portevin et al. (16). This ratio is based on the MFI of CD27 in CD4^+^ T-cells over MFI of CD27 in IFN-γ^+^CD4^+^ T-cells. **(A)** CD27 MFI ratio after PPD or **(B)** ESAT-6/CFP-10 stimulation in patients with active TB and LTBI individuals. Horizontal lines represent medians. Differences between conditions were calculated using the two-tailed Mann-Whitney *U*-test. ****p < 0.0001. **(C,D)** Correlation of the percentage of CD27^−^ marker within IFN-γ^+^CD4^+^ specific T-cells with the CD27 MFI ratio after PPD or ESAT-6/CFP-10 stimulation. Correlation was calculated using the two-tailed non-parametric Spearman test. aTB, active TB; LTBI, latent tuberculosis infection.

An approach based on CD27 MFI on CD4^+^ T-cells was assessed as suggested by Portevin et al. (16). This method consists on evaluating the ratio between CD27 MFI in CD4^+^ T-cells and the MFI of CD27 in specific IFN-γ^+^CD4^+^ T-cells. Therefore, a low CD27 MFI on specific CD4^+^ T-cells which produce IFN-γ^+^ implies a high CD27 ratio (this is a consequence of an increase of the CD27^−^IFN-γ^+^CD4^+^ T-cells phenotype). In this study, a high ratio was significantly associated with active TB when T-cells were stimulated with PPD (Figure 3A; *p* < 0.0001) or ESAT-6/CFP-10 (Figure 3B; *p* < 0.0001).

In order to explore whether a high CD27 MFI ratio was associated with an increase of the percentage of CD27^−^ within IFN-γ^+^CD4^+^ T-cells, a Spearman test correlation was performed. A positive correlation between these two variables was observed in T-cells responding to PPD (Figure 3C) or ESAT-6/CFP-10 (Figure 3D), which is supported by a significant correlation coefficient (for PPD: Spearman's rho = 0.869, *p* < 0.0001; for ESAT-6/CFP-10: Spearman's rho = 0.892, *p* < 0.0001).

The approach based on CD27 MFI was also assessed on (i) TNF-α^+^CD4^+^ T-cells, (ii) IFN-γ^+^TNF-α^+^CD4^+^ T-cells, and (iii) IFN-γ^+^ and/or TNF-α^+^CD4^+^ T-cells after *M. tuberculosis* specific stimulation. These ratios were significantly higher in active TB patients than in LTBI individuals after PPD (Figure S5A in Supplementary Material) or ESAT-6/CFP-10 (Figure S5B in Supplementary Material) stimulation.

### Diagnostic Accuracy of the Different Biomarkers

**Figure 4:**
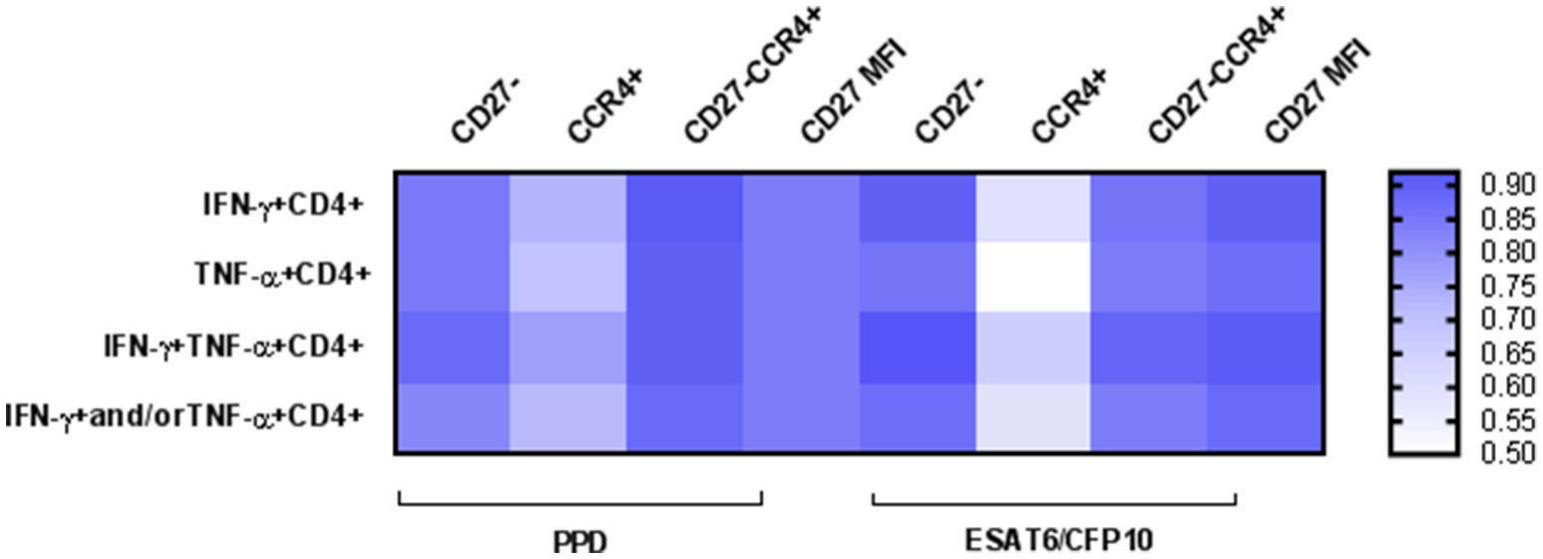
Heatmap depicting Areas Under the Curve (AUC) values for the different approaches. A ROC curve analysis was performed to determine the diagnostic accuracy for TB diagnosis. AUC values are represented for: (i) percentage of CD27^−^ marker, (ii) percentage of CCR4^+^ marker, (iii) percentage of CD27^−^CCR4^+^ signature, and (iv) CD27 MFI ratio. Values are shown for the different CD4^+^ T-cells functional populations analyzed after PPD or ESAT-6/CFP-10 specific stimulation. High AUC values are indicated by intensity of blue color.

To asses TB diagnostic accuracy of the different approaches analyzed in this study, we performed a ROC curve analysis (Table 2). Highest AUC values (AUC > 0.90) for discriminating active TB from LTBI were achieved when evaluating: (i) CD27 within IFN-γ^+^CD4^+^ and IFN-γ^+^TNF-α^+^ CD4^+^ T-cells in response to ESAT-6/CFP-10 [AUC (95% confidence interval, CI) 0.90 (0.79–1.01) and 0.92 (0.84–1.01) respectively], (ii) CD27 and CCR4 markers together within IFN-γ^+^CD4^+^, TNF-α^+^CD4^+^ and IFN-γ^+^TNF-α^+^ CD4^+^ T-cells in response to PPD [AUC (95% CI) 0.91 (0.83–0.99), 0.90 (0.82–0.99), and 0.90 (0.81–0.99) respectively], and (iii) CD27 MFI ratio performed on IFN-γ^+^CD4^+^ and IFN-γ^+^TNF-α^+^CD4^+^ T-cells after ESAT-6/CFP-10 specific stimulation [AUC (95% CI) 0.90 (0.79–1.01) and 0.91 (0.82–1.01), respectively]. The lowest diagnostic accuracy was observed when CCR4 marker was evaluated alone (Figure 4).

**Table 2:** ROC curve analysis of the different approaches.

**PHENOTYPE**	**AUC (95% CI)**	
	**PPD**	**ESAT-6/CFP-10**
**CD27**^**−**^		
IFNγ^+^CD4^+^	0.85 (0.72–0.97)	0.90 (0.79–1.01)
TNF-α^+^CD4^+^	0.85 (0.74–0.97)	0.86 (0.75–0.97)
TNF-α^+^IFN-γ^+^CD4^+^	0.88 (0.77–098)	0.92 (0.84–1.01)
IFNγ^+^and/orTNF-α^+^CD4^+^	0.82 (0.69–0.95)	0.87 (0.76–0.98)
**CCR4**^**+**^		
IFNγ^+^CD4^+^	0.73 (0.59–0.87)	0.60 (0.43–0.78)
TNF-α^+^CD4^+^	0.70 (0.55–0.85)	0.50 (0.33–0.67)
TNF-α^+^IFN-γ^+^CD4^+^	0.77 (0.64–0.91)	0.65 (0.49–0.83)
IFNγ^+^and/orTNF-α^+^CD4^+^	0.72 (0.58–0.87)	0.59 (0.43–0.75)
**CD27**^**−**^**CCR4**^**+**^		
IFNγ^+^CD4^+^	0.91 (0.83–0.99)	0.86 (0.73–0.98)
TNF-α^+^CD4^+^	0.90 (0.82–0.99)	0.84 (0.71–0.96)
TNF-α^+^IFN-γ^+^CD4^+^	0.90 (0.81–0.99)	0.89 (0.77–0.99)
IFNγ^+^and/orTNF-α^+^CD4^+^	0.88 (0.78–0.97)	0.84 (0.71–0.96)
**MFI CD27**		
IFNγ^+^CD4^+^	0.84 (0.71–0.97)	0.90 (0.79–1.01)
TNF-α^+^CD4^+^	0.84 (0.71–0.97)	0.87 (0.76–0.97)
TNF-α^+^IFN-γ^+^CD4^+^	0.84 (0.71–0.97)	0.91 (0.82–1.01)
IFNγ^+^and/orTNF-α^+^CD4^+^	0.84 (0.71–0.97)	0.88 (0.77–0.99)

*AUC, area under the curve; CI, confidence interval; PPD, purified protein derivative*.

## Discussion

Approaches based on the study of the host immune response have emerged as potential tools for TB management, studying the interplay between the host and *M. tuberculosis*, and discovering suitable disease biomarkers. Here we have analyzed specific immune-mechanisms based on the characterization of different T-cell subsets and the expression of surface receptors such as CD27 and/or CCR4 involved in the migration of certain lymphocytes to the disease inflammatory sites. Briefly, our results confirm previous reports on CD27 modulation in specific CD4^+^ T-cells producing IFN-γ, which is downregulated during active disease. Furthermore, a high CD27 MFI ratio proposed as a disease biomarker by other authors (16, 17) was associated with active TB patients compared to LTBI individuals. This study adds novel information on other potential homing biomarkers such as CCR4, showing that in combination with CD27 (CD27^−^CCR4^+^IFN-γ^+^CD4^+^ T-cells) could be a phenotype to discriminate between disease and infection. Furthermore, we also characterized CD27 on CD4^+^ T-cells producing TNF-α, observing that this marker has a high power of discrimination when analyzed within IFN-γ^+^TNF-α^+^CD4^+^ T-cells population.

The study of cytokine profiles on specific *M. tuberculosis* T-cell responses has suggested that specific subsets may serve as disease biomarkers associated with bacterial load, treatment response or disease outcome. This data is still limited and need to be further evaluated. In this context, a previous study indicated that CD4^+^ T-cells that only produce TNF-α could be associated with active disease (23). Others have reinforced this data suggesting that PPD specific TNF-α^+^ CD4^+^ T-cells with an effector phenotype can accurately discriminate active TB from LTBI, or even recently acquired from remote LTBI (24, 25). The results we obtained on *M. tuberculosis* T-cells functional profile were also in agreement with those obtained in previous studies, as we found that CD4^+^ T-cell only producing TNF-α in response to PPD were increased in active TB patients. While it is widely accepted that CD4^+^ T-cells play an essential role against the bacilli's immune response, protective immunity to *M. tuberculosis* by CD8^+^ T-cells still remains controversial. In the last years, CD8^+^ T-cells have emerged as a possible population actively involved in the immunopathology (26–28). Our study also corroborates the importance of CD8^+^ T-cells as TB control players, showing detectable cytokine responses in this population which tend to be higher during disease in response to ESAT-6 and CFP-10 antigens. One important challenge for TB management and diagnosis is to find specific antigens capable to elicit CD8^+^ T-cells responses. In this context, a new generation of QFN called QFN-Plus has incorporated new peptides able to induce IFN-γ responses on CD4^+^ and CD8^+^ T-cells, trying to increase the accuracy of the assay and to correlate T-cell responses with antigen load or high risk of TB progression (29). However, data about its accuracy over classical IGRAs or correlation with disease state is still limited.

In this study, we confirm that the evaluation of the frequency of CD27^−^ within functional CD4^+^ T-cells, together with the CD27 MFI ratio, were suitable biomarkers for TB which could discriminate disease from infection with acceptable AUC values between 0.82 and 0.92 depending on the stimuli used (PPD or ESAT-6/CFP-10). The CD27 marker is a member of the TNF-receptor superfamily, expressed by lymphocytes, which is downregulated during effector differentiated T-cells able to produce cytokines (14). Thus, due to the persistent antigenic stimulation during active TB, it has been proposed as an immune biomarker of the disease (16, 17, 30, 31). The recently developed immune assay based on the detection of CD27 MFI ratio (TAM-TB assay) has also been proposed as an alternative way for measuring this receptor (16). Here, we show that the percentage of CD27^−^ significantly correlated with CD27 MFI quantification, indicating that both immune strategies are accurate enough for TB diagnosis. However, the calculation of a ratio based on MFI allows normalization of the results avoiding subjectivity and discrepancies on CD27 positive or negative gating. In addition, our results also add new information about the CCR4 homing marker. We have observed that overexpression of CCR4 receptor within IFN-γ^+^CD4^+^ T-cells is a poor immune biomarker of disease when evaluated alone, however, when combined together with CD27^−^IFN-γ^+^CD4^+^ T-cells, its discriminatory capacity increased (0.91 after PPD stimulation). CCR4 has been suggested as a lung homing receptor expressed on T-cells. A recent study focused on the detection of CD27 or CCR4 markers (among others) on active TB and LTBI individuals (with and without HIV infection), found that in an active TB context T-cells presented a CD27 marker downregulation. In contrast, no difference on CCR4 expression was found regarding the clinical status of the individuals (irrespective of HIV infection) when this marker was detected alone (31). In addition, other possible disease immune markers have been studied by others in order to improve TB diagnosis accuracy. For example, the expression of the activation marker HLA-DR on specific CD4^+^ T-cells has shown a good discriminatory capacity between active TB and LTBI (30, 31). This study also adds new data on CD27 and/or CCR4 characterization within T-cells secreting IFN-γ and/or TNF-α. IFN-γ cytokine does not fully represent the response against *M. tuberculosis*, having TNF-α an important role during active TB disease. In this context, we found that CD27^−^ and/or CCR4^+^ expression within: (i) TNF-α^+^CD4^+^; (ii) IFN-γ^+^TNF-α^+^CD4^+^ T-cells, and (iii) IFNγ^+^and/orTNF-α^+^CD4^+^ T-cells, as well as CD27 MFI ratio measured in these functional populations, were increased in active TB patients in comparison with LTBI individuals. Highest discriminatory capacities were achieved when measuring CD27^−^ or CD27 MFI ratio within IFN-γ^+^TNF-α^+^CD4^+^ T-cells (AUC 0.92 and 0.91 after ESAT-6/CFP-10 stimulation). This indicates that CD4^+^ T-cells lacking CD27 marker are able to differentiate into effector T-cells and increment their capacity to secrete IFN-γ and/or TNF-α cytokines. In addition, the study of CD27 on TNF-α producing T-cells increased the detection of positive responses by flow cytometry after ESAT-6/CFP-10 stimulation, especially in the LTBI group.

In mice it has been shown that IFN-γ^+^CD4^+^ T-cells which have CD27 receptor downregulated are accumulated preferentially in the lungs during mycobacterial infection (32). Furthermore, CD27^low^ specific CD4^+^ T-cells are increased in lungs of patients with active TB, and percentages of this subset are higher in lung tissue than in blood. Interestingly, when this T-cell subset was detected in blood, it correlated with tissue destruction and TB severity. This correlation was not observed when the T-cell subset was detected in the lungs. The reasons of this discordance are still unclear, but could be explained by its generation from different precursors. CD27^low^ specific CD4^+^ T-cells can be generated in the lymph nodes and then migrate to peripheral blood, while those located in the lungs can be generated locally from other precursors (14). In the same context, severe TB induced the upregulation of CCR4 gene (among others) in pulmonary compartments of infected rhesus monkeys (33). According to these findings, it would be interesting to study CD27 and/or CCR4 in samples from active TB patients coming from the site of infection in order to understand better the mechanisms and pathogenesis of the disease. Further studies in this direction need to be addressed.

The monitoring of anti-TB therapy efficacy is a key point for TB control. Petruccioli E. et al. showed that the expression of CD27 increases on specific T-cells in cured active TB patients after 1 year of therapy completion (17). These findings suggest that CD27 might serve as a tool for following-up active TB patients, detecting efficacy of treatment, and exploring inflammatory status. In this line, the study of a differential phenotype on T-cells expressing CD27 and/or CCR4 homing markers during the treatment follow-up of active TB patients is needed for validating these findings. Some data support that only after 2 months of TB therapy the expression of CD27 starts its modulation (14). Thus, according to these data, the detection of CD27^−^CD4^+^ T-cells during the first month of treatment on patients recruited in our study should not be altered. This hypothesis is also reinforced by our results, as no significant correlation between days of treatment (within the firsts 4 weeks of therapy) and the CD27/CCR4 expression was found.

Limitations of this study need to be addressed. First, although results obtained on CD27 are robust among different studies, it is important to uniformly validate this immune assay for routine purposes and results reproducibility, choosing common starting material (PBMCs or whole blood), same specific stimuli and fluorochromes, as well as standardizing protocols. And second, the triggering of host immune responses and disease outcome does not depend only on a single factor. Immune status of the host depends on a *troika* of multiple parameters covering host genetics, the pathogen and extrinsic elements (34). Therefore, it is necessary to study and combine all these variables together in other to translate possible host immune TB biomarkers into potential immune assays with clinical applications.

In summary, our findings on surface homing markers such as CD27 and CCR4 on *M. tuberculosis* specific CD4^+^ T-cells gather the required features for using them as potential TB biomarkers. Therefore, it would be crucial to further evaluate these receptors in a larger cohort of patients in order to develop possible and simplified routine immune assays for TB diagnosis, assessment of therapy efficacy/relapse, and risk-stratification of LTBI individuals.

## Ethics Statement

The study was approved by the Ethics Committee of the Hospital Germans Trias i Pujol (Reference number PI-17-134). All enrolled subjects gave a written informed consent for participating in this study.

## Author Contributions

IL and JD designed the study. IL, MF-S, and RV-H designed the experiments. BM-M, SV, RV-H, and IL performed the experiments. MDS-G, ZS, MJ-F, CC, JR-M, J-PM, IM-P, YG-D, LL-C, JS, and CP contributed with resources. IL, MF-S, and RV-H analyzed the data. IL, JD, AL, and CP supervised the study. IL and JD wrote the paper. All authors revised and approved the manuscript.

### Conflict of Interest Statement

The authors declare that the research was conducted in the absence of any commercial or financial relationships that could be construed as a potential conflict of interest.

## Supplementary Material

Figure S1 Gating strategy used for CD4^+^/CD8^+^ T-cells cytokine secretion and CD27/CCR4 analysis. Aggregated cells were taken off by gating on the diagonal that appears with Forward-Scatter (height; FSC-H) vs. Forward-Scatter (area; FSC-A) dot plot. For CD4^+^ and CD8^+^ T-cells gating, alive CD3^+^ T-cells were first selected. Cytokine profiles on T-cells were analyzed on CD4^+^/CD8^+^ T-cells. CD27/CCR4 expression was studied within IFN-γ^+^CD4^+^ T-cells gated from alive CD3^+^ T-cells.

Figure S2 Correlation of homing markers expression with days of treatment. Correlation of days after starting anti-TB therapy in active TB patients (within the 4 weeks of starting treatment) with **(A)** percentage of CD27^−^ and/or CCR4^+^ within IFN-γ^+^CD4^+^ T-cells in response to PPD or **(B)** ESAT-6/CFP-10 antigens. Correlation was calculated using the two-tailed non-parametric Spearman test.

Figure S3 CD27^−^ and/or CCR4^+^ phenotype within functional CD4^+^ T-cells producing IFN-γ and/or TNF-α in patients with active TB and LTBI individuals. Percentage of PPD or ESAT-6/CFP-10 specific CD27^−^, CCR4^+^, and CD27^−^CCR4^+^ within **(A)** TNF-α^+^CD4^+^ T-cells, **(B)** IFN-γ^+^TNF-α^+^CD4^+^ T-cells, and **(C)** IFN-γ^+^ and/or TNF-α^+^CD4^+^ T-cells. Horizontal lines represent medians. Differences between conditions were calculated using the two-tailed Mann-Whitney *U*-test. ^*^*p* < 0.05, ^**^*p* < 0.01, ^***^*p* < 0.001, ^****^*p* < 0.0001. ns, non-significant; aTB, active TB; LTBI, latent tuberculosis infection.

Figure S4 Relationship of the CD27^−^ expression on the different antigen-specific T-cells populations analyzed. Correlation of the CD27^−^ expression on IFN-γ^+^CD4^+^ T-cells with **(A)** TNF-α^+^CD4^+^ or **(B)** TNF-α^+^IFN-γ^+^ CD4^+^ T-cells after PPD or ESAT-6/CFP-10 antigen stimulation. Correlation was calculated using the two-tailed non-parametric Spearman test.

Figure S5 CD27 MFI ratio calculated on functional CD4^+^ T-cells producing IFN-γ and/or TNF-α. A ratio based on CD27 MFI was calculated after specific stimulation in active TB patients and LTBI individuals. This ratio is based on the MFI of CD27 in CD4^+^ T-cells over: (i) MFI of CD27 in TNF-α^+^CD4^+^ T-cells, (ii) MFI of CD27 in IFN-γ^+^TNF-α^+^CD4^+^ T-cells, and (iii) MFI of CD27 in IFN-γ^+^ and/or TNF-α^+^CD4^+^ T-cells after **(A)** PPD or **(B)** ESAT-6/CFP-10 antigen stimulation. Horizontal lines represent medians. Differences between conditions were calculated using the two-tailed Mann-Whitney *U*-test. ^****^*p* < 0.0001. aTB, active TB; LTBI, latent tuberculosis infection.
