# Supplementary figures and images for: Study of CD27 and CCR4 Markers on Specific CD4+ T-Cells as Immune Tools for Active and Latent Tuberculosis Management
Source: Front Immunol. 2019 Jan 9;9:3094. doi: 10.3389/fimmu.2018.03094 (PMC6334476; doi:10.3389/fimmu.2018.03094)

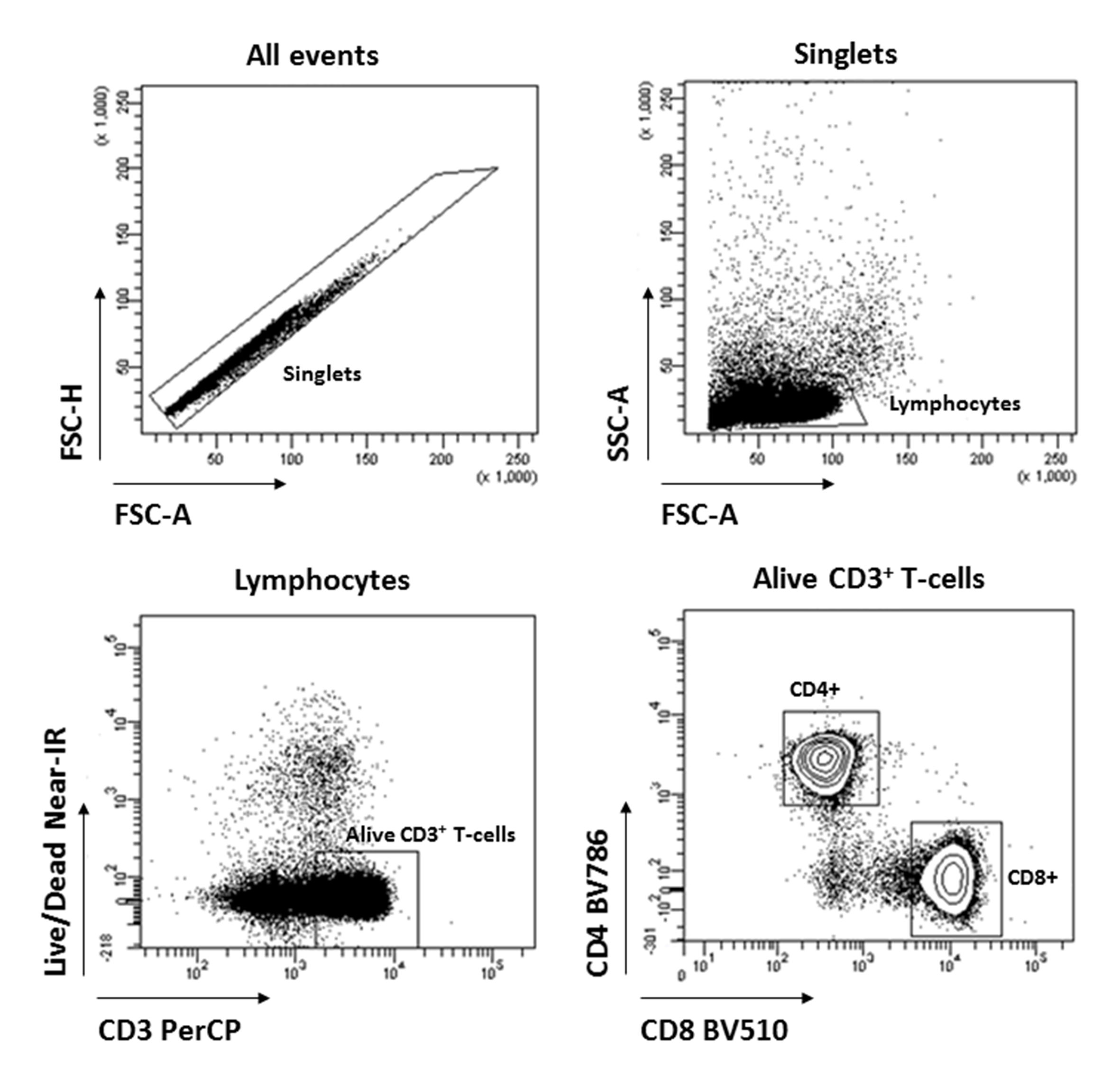

Supplement: Figure S1 — Gating strategy used for CD4+/CD8+ T-cells cytokine secretion and CD27/CCR4 analysis. Aggregated cells were taken off by gating on the diagonal that appears with Forward-Scatter (height; FSC-H) vs. Forward-Scatter (area; FSC-A) dot plot. For CD4+ and CD8+ T-cells gating, alive CD3+ T-cells were first selected. Cytokine profiles on T-cells were analyzed on CD4+/CD8+ T-cells. CD27/CCR4 expression was studied within IFN-γ+CD4+ T-cells gated from alive CD3+ T-cells. [file Image_1.TIF]

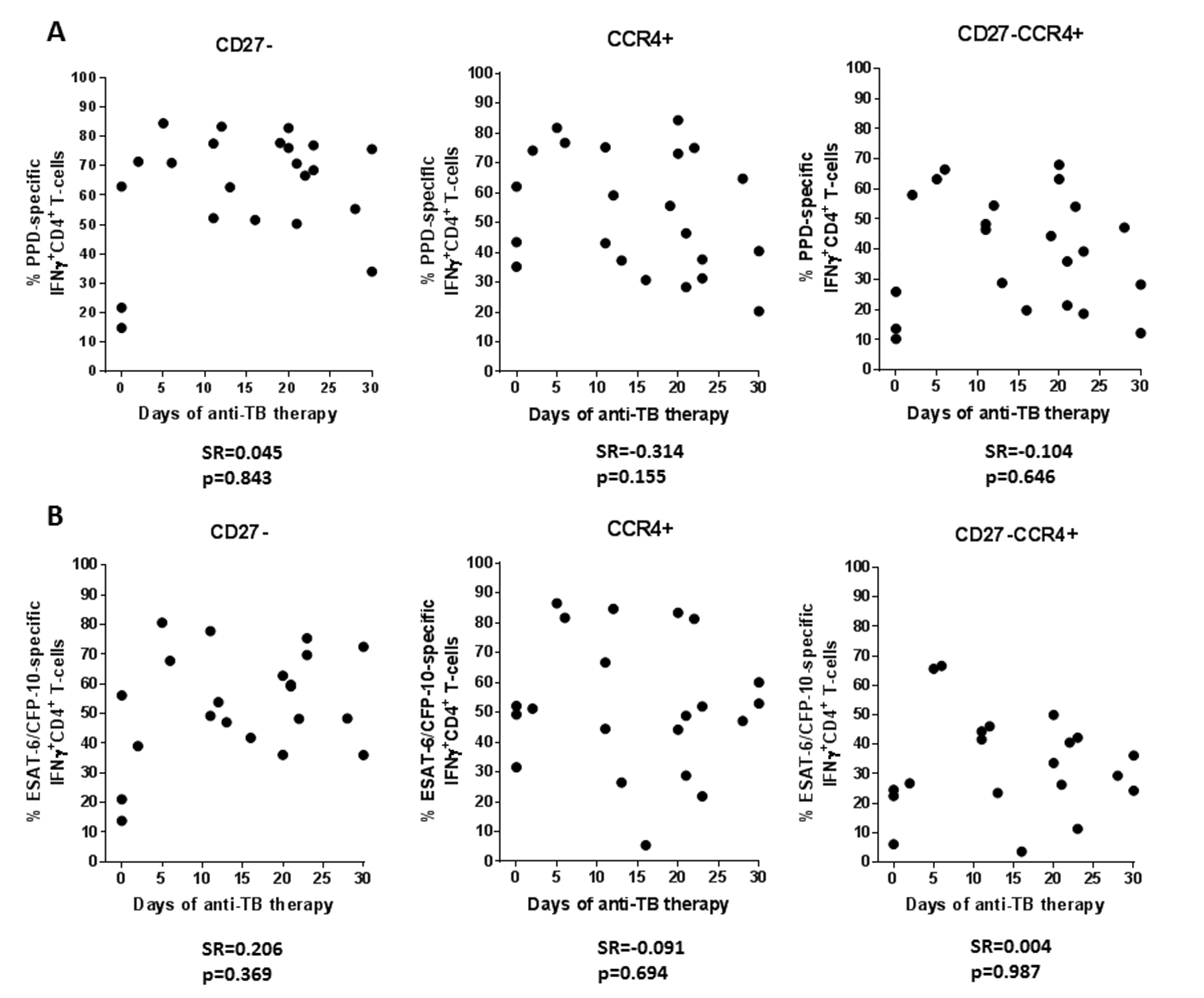

Supplement: Figure S2 — Correlation of homing markers expression with days of treatment. Correlation of days after starting anti-TB therapy in active TB patients (within the 4 weeks of starting treatment) with (A) percentage of CD27− and/or CCR4+ within IFN-γ+CD4+ T-cells in response to PPD or (B) ESAT-6/CFP-10 antigens. Correlation was calculated using the two-tailed non-parametric Spearman test. [file Image_2.TIF]

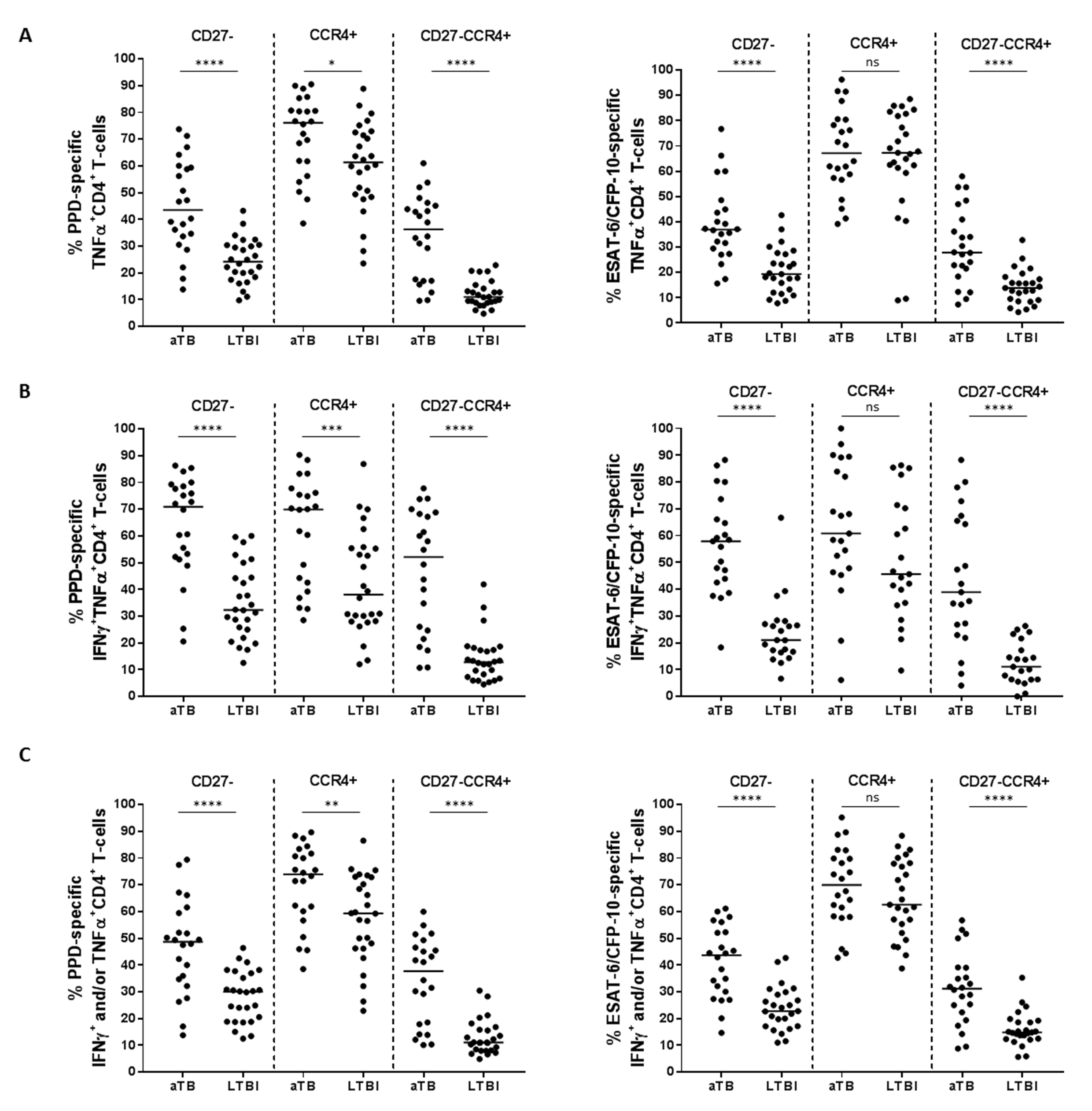

Supplement: Figure S3 — CD27− and/or CCR4+ phenotype within functional CD4+ T-cells producing IFN-γ and/or TNF-α in patients with active TB and LTBI individuals. Percentage of PPD or ESAT-6/CFP-10 specific CD27−, CCR4+, and CD27−CCR4+ within (A) TNF-α+CD4+ T-cells, (B) IFN-γ+TNF-α+CD4+ T-cells, and (C) IFN-γ+ and/or TNF-α+CD4+ T-cells. Horizontal lines represent medians. Differences between conditions were calculated using the two-tailed Mann-Whitney U-test. *p < 0.05, **p < 0.01, ***p < 0.001, ****p < 0.0001. ns, non-significant; aTB, active TB; LTBI, latent tuberculosis infection. [file Image_3.tif]

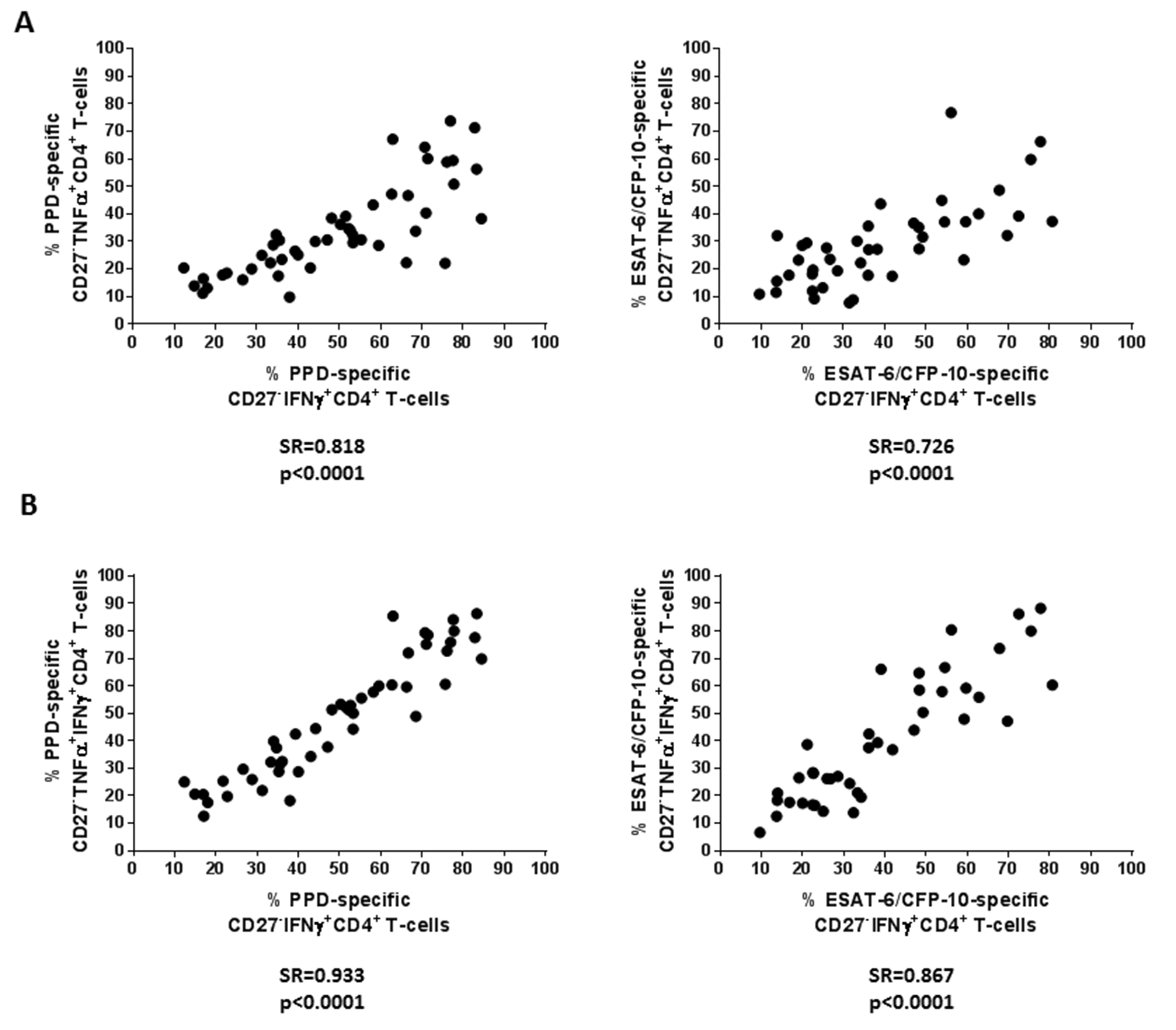

Supplement: Figure S4 — Relationship of the CD27− expression on the different antigen-specific T-cells populations analyzed. Correlation of the CD27− expression on IFN-γ+CD4+ T-cells with (A) TNF-α+CD4+ or (B) TNF-α+IFN-γ+ CD4+ T-cells after PPD or ESAT-6/CFP-10 antigen stimulation. Correlation was calculated using the two-tailed non-parametric Spearman test. [file Image_4.TIF]

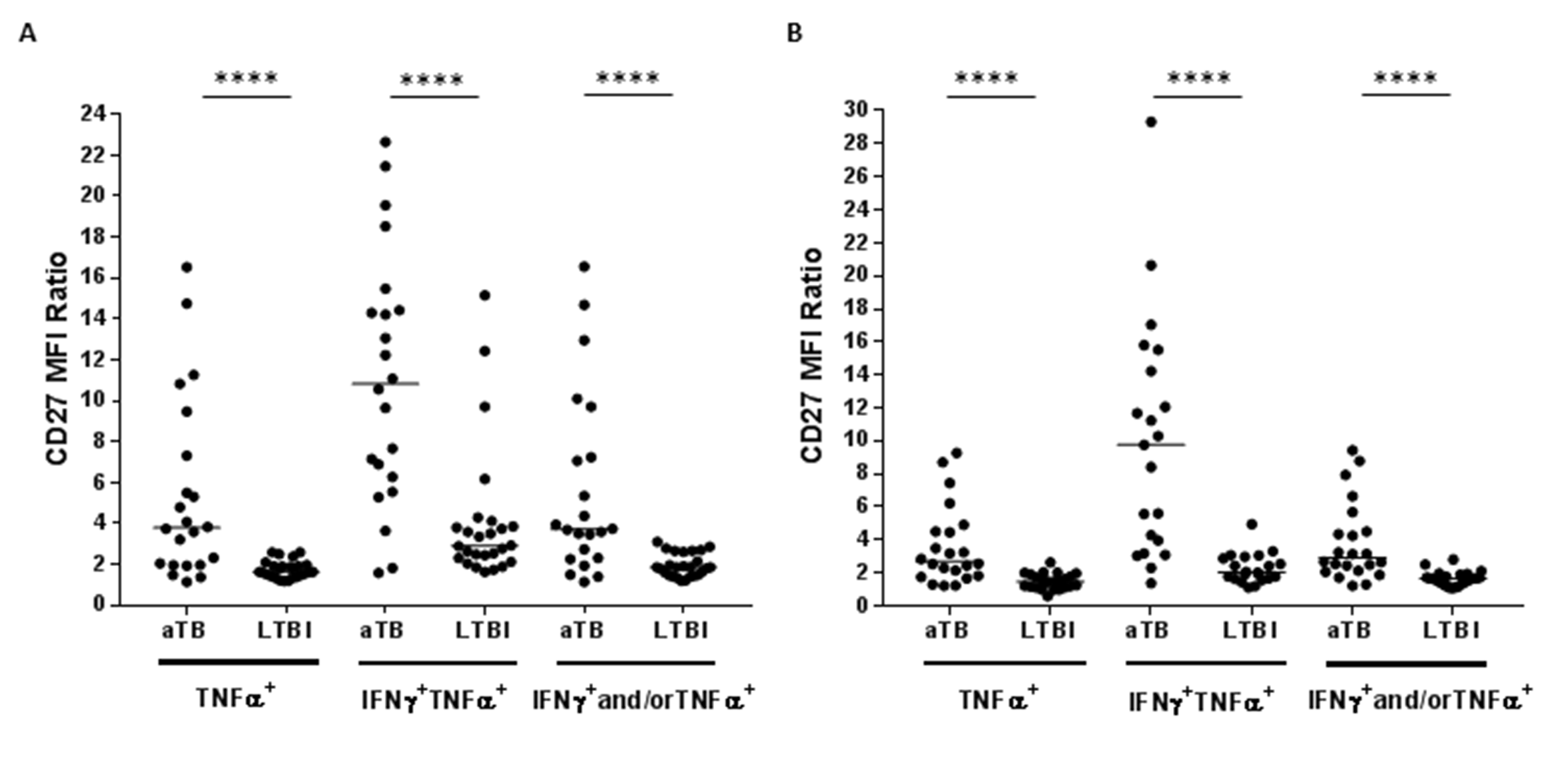

Supplement: Figure S5 — CD27 MFI ratio calculated on functional CD4+ T-cells producing IFN-γ and/or TNF-α. A ratio based on CD27 MFI was calculated after specific stimulation in active TB patients and LTBI individuals. This ratio is based on the MFI of CD27 in CD4+ T-cells over: (i) MFI of CD27 in TNF-α+CD4+ T-cells, (ii) MFI of CD27 in IFN-γ+TNF-α+CD4+ T-cells, and (iii) MFI of CD27 in IFN-γ+ and/or TNF-α+CD4+ T-cells after (A) PPD or (B) ESAT-6/CFP-10 antigen stimulation. Horizontal lines represent medians. Differences between conditions were calculated using the two-tailed Mann-Whitney U-test. ****p < 0.0001. aTB, active TB; LTBI, latent tuberculosis infection. [file Image_5.TIF]
